# Micropearls and other intracellular inclusions of amorphous calcium carbonate: an unsuspected biomineralization capacity shared by diverse microorganisms

**DOI:** 10.1111/1462-2920.15498

**Published:** 2021-05-06

**Authors:** Inés Segovia‐Campos, Agathe Martignier, Montserrat Filella, Jean‐Michel Jaquet, Daniel Ariztegui

**Affiliations:** ^1^ Department of Earth Sciences University of Geneva Geneva CH‐1205 Switzerland; ^2^ Department F.‐A. Forel University of Geneva Geneva CH‐1205 Switzerland

## Abstract

An unsuspected biomineralization process, which produces intracellular inclusions of amorphous calcium carbonate (ACC), was recently discovered in unicellular eukaryotes. These mineral inclusions, called *micropearls*, can be highly enriched with other alkaline‐earth metals (AEM) such as Sr and Ba. Similar intracellular inclusions of ACC have also been observed in prokaryotic organisms. These comparable biomineralization processes involving phylogenetically distant microorganisms are not entirely understood yet. This review gives a broad vision of the topic in order to establish a basis for discussion on the possible molecular processes behind the formation of the inclusions, their physiological role, the impact of these microorganisms on the geochemical cycles of AEM and their evolutionary relationship. Finally, some insights are provided to guide future research.

## Introduction

Biomineralization is a biologically controlled process by which living organisms form minerals from the selective extraction of certain chemical elements present in their environment (Mann, 2001; Bazylinski and Frankel, 2003). Some authors also include in the definition of biomineralization the biologically induced mineralization, where the metabolic activity of some organisms changes the surrounding environmental conditions and triggers mineral precipitation (Weiner and Dove, 2003).

A large number of organisms form minerals (from prokaryotes to pluricellular eukaryotes) and this process can take place at different locations: (i) in the extracellular space (outside the cell), (ii) in the intercellular space (between cells), (iii) in the epicellular space (on the cell walls) or (iv) in the intracellular space (inside the cells) (Mann, 2001). For simplicity, the epicellular and intercellular spaces can be considered as extracellular locations.

In microorganisms, the chemical composition and the physiological function of biominerals vary depending on the group of organisms forming them. For instance, magnetotactic bacteria form intracellular structures called magnetosomes, that are composed of magnetite (Fe_3_O_4_) or greigite (Fe_3_S_4_), acting as geomagnetic field sensors (Schüler and Frankel, 1999), whereas diatoms present silica (SiO_2_ · nH_2_O) shells, known as frustules, that act as exoskeletons and confer protection against mechanical stress and UV light (Hamm *et al*., 2003; Ingalls *et al*., 2010).

Calcium carbonate biominerals are the most abundant and widespread minerals among organisms (Weiner and Dove, 2003). There are five main polymorphic biogenic varieties of calcium carbonate in descending order of thermodynamic stability at ambient conditions: calcite, aragonite, vaterite, monohydrocalcite and amorphous calcium carbonate (ACC) (Levi‐Kalisman *et al*., 2002; Addadi *et al*., 2003; Weiner and Dove, 2003). While the first three minerals are anhydrous crystalline polymorphs, monohydrocalcite is a hydrated crystalline mineral. According to Addadi *et al*. (2003), ACC is the least stable polymorph, including both hydrated and anhydrous forms (called *stable* and *transient* forms respectively).

Several strains of cyanobacteria are known to be involved in the extracellular formation of microbial carbonates, called microbialites (Burne and Moore, 1987; Riding, 2000). Indeed, the photosynthetic activity of these prokaryotic organisms modifies the surrounding environmental conditions by rising the alkalinity, which triggers the extracellular precipitation of calcium carbonate (Verrecchia *et al*., 1995; Arp *et al*., 2001; Ludwig *et al*., 2005; Liang *et al*., 2013).

Foraminifera and coccolithophores are the major unicellular eukaryotes (protists) that produce calcium carbonate to form respectively, epicellular shells and scales that act as protective structures (Mann, 2001; Madigan *et al*., 2010; Raven and Knoll, 2010). In foraminifera, the shells can be composed of either calcite or aragonite, while coccolithophores only present calcite scales called coccoliths. Some studies suggest that Ca and carbonate destined to mineralize the shell of foraminifera may be previously stored in intracellular pools as ACC (Erez, 2003; Bentov and Erez, 2006; Khalifa *et al*., 2016), but such inclusions have never been described. On the other hand, the formation of coccoliths is an entirely intracellular process (Brownlee *et al*., 2021) and vacuole‐like‐compartments containing a phosphorous‐rich disordered calcium phase have been observed (Sviben *et al*., 2016).

Apart from coccolithophores and a few isolated cases, such as the giant sulfur bacterium *Achromatium* (class Gammaproteobacteria) (Head *et al*., 2000; Gray, 2006; Isambert *et al*., 2007; Taoka *et al*., 2014; Schorn *et al*., 2020), the formation of calcium carbonate by microorganisms has traditionally been considered an extracellular process, the detection of intracellular ACC being extremely rare. Only a few forgotten cases of intracellular ACC inclusions had been reported in some marine and freshwater ciliates in the past (Fauré‐Fremiet and Gauchery, 1957).

In recent years, several studies have revealed the ability of some phylogenetically distant microorganisms to form intracellular ACC inclusions that can contain other alkaline‐earth metals (AEM): from cyanobacteria (Couradeau *et al*., 2012; Benzerara *et al*., 2014) and magnetotactic Alphaproteobacteria (Monteil *et al*., 2020) to unicellular eukaryotes (Martignier *et al*., 2017). In addition, it has been shown that the intracellular mineral inclusions of the giant sulfur bacterium *Achromatium*, which have been commonly described as calcite bodies, are actually composed of ACC (Benzerara *et al*., 2020).

In unicellular eukaryotes, these inclusions, called *micropearls*, present internal concentric zonations due to frequent slight variations in their chemical composition (Martignier *et al*., 2017). Interestingly, similar concentric structures have also been described in the intracellular ACC inclusions of several cyanobacteria (Cam *et al*., 2016). However, the physiological function of these inclusions, as well as the molecular mechanisms involved in their formation in both eukaryotic and prokaryotic organisms are poorly known to date.

This review aims to compile the existing information about the intracellular ACC biomineralization process in microorganisms as a basis for discussion on the molecular mechanisms involved, the biological role of intracellular ACC, the impact of these organisms on the environment and their possible evolutionary relationships. Finally, we also suggest future research directions to achieve a deeper understanding of the topic.

## Intracellular ACC inclusions in protists (micropearls)

In 2013, different types of intracellular Ba and Sr enriched ACC inclusions were observed in eukaryotic organisms for the first time in Lake Geneva (Switzerland, France) during a routine examination of plankton using scanning electron microscopy (SEM) (Jaquet *et al*., 2013). This finding was the first evidence of an unexpected intracellular ACC biomineralization process in protists. The mineral inclusions appeared as bright spheres of diameters between 0.6 and 3 μm, and several types were differentiated according to their composition. Ba/Ca and Sr/Ca atomic ratios of the micropearls were respectively, measured to be up to 4800 and 10 times higher than in the surrounding lake water (Jaquet *et al*., 2013).

**Fig 1:**
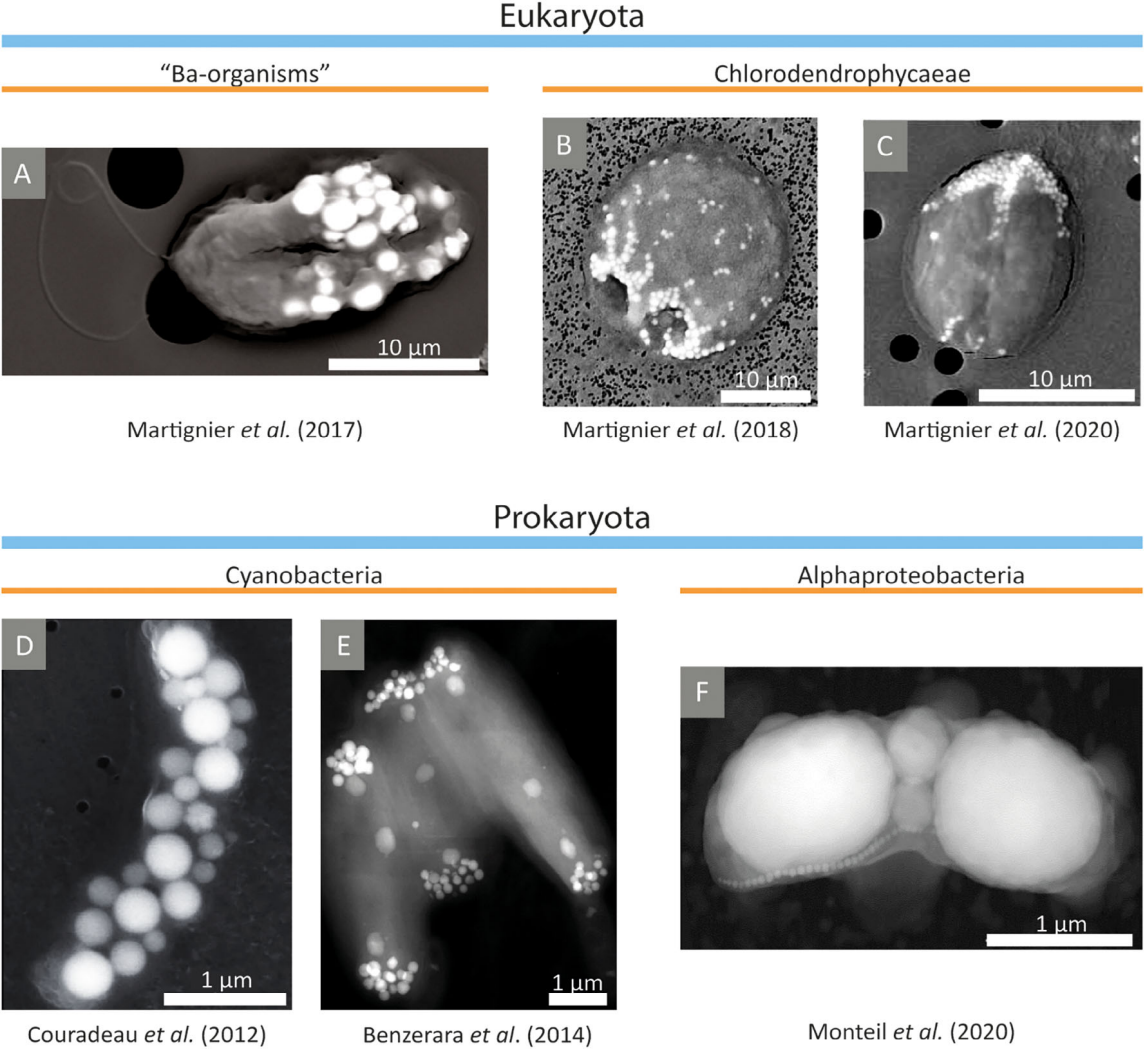
Electron microscopy images of diverse microorganisms presenting ACC inclusions. They appear as white granules due to their electron‐dense properties and relatively high atomic mass. (A) SEM (in backscattered electron mode) image of the undetermined microorganism from Lake Geneva containing Ba‐rich inclusions. Two flagella can be observed emerging from the left pole of the cell. (B, C) SEM images of Chlorodendrophyceae algae: (B) ACC inclusions in *Tetraselmis cordiformis* are spherical and mainly occupy the anterior part of the cell while (C) the inclusions in *Scherffelia dubia* are mainly located in the posterior pole of the cell. (D) SEM image of a *Gloeomargarita lithophora* cell presenting ACC inclusions scattered in the cytoplasm. (E) STEM‐HAADF image of a *Candidatus* Synechococcus calcipolaris cell showing ACC inclusions clustered at the cell poles. The largest and faintest inclusions located in the longitudinal axis of the cell correspond to polyphosphate granules. (F) STEM‐HAADF image of the magnetotactic bacterium sampled in Lake Pavin showing two large ACC inclusions and two small inclusions at the septation site of the dividing cell. Magnetosomes are the smaller inclusions aligned at one side of the cell forming a chain. The images are adapted with permission from: (A) Martignier *et al*. (2017). Copyright 2016 John Wiley & Sons (B) Martignier *et al*. (2018). Copyright 2018 Martignier, Filella, Pollok, Melkonian, Bensimon, Barja, Langenhorst, Jaquet, and Ariztegui. (C) Martignier *et al*. (2020). Copyright 2020 Elsevier. (D) Couradeau *et al*. (2012) with permission from AAAS. (E) Benzerara *et al*. (2014). Copyright 2014 Benzerara, Skouri‐Panet, Li, Férard, Gugger, Laurent, Couradeau, Ragon, Cosmidis, and Menguy. (F) Monteil *et al*. (2020). Copyright 2020 Monteil, Benzerara, Menguy, Bidaud, Michot‐Achdjian, Bolzoni, Mathon, Coutaud, Alonso, Garau, Jézéquel, Duprat, Guyot, and Lefevre.

Further investigations confirmed that the inclusions, known as micropearls, are produced in the intracellular space of certain protists and their chemical composition is specific to the different organisms forming them (Martignier *et al*., 2017). Microorganisms producing Ba‐rich micropearls, called here ‘Ba‐organisms’, are still undetermined (Fig. 1A); however, the green microalga *Tetraselmis cordiformis* (class Chlorodendrophyceae) has been identified as the organism forming Sr‐rich ACC inclusions in Lake Geneva (Martignier *et al*., 2017). Micropearls were also found in cultured strains of *T*. *cordiformis* obtained from algae collections (Fig. 1B). More recently, ACC inclusions have been found to be a widespread trait within the class Chlorodendrophyceae (Martignier *et al*., 2018, 2020), composed of two genera: *Tetraselmis* (mainly including marine species) and *Scherffelia* (including a single freshwater species) (Fig. 1C).

**Fig 2:**
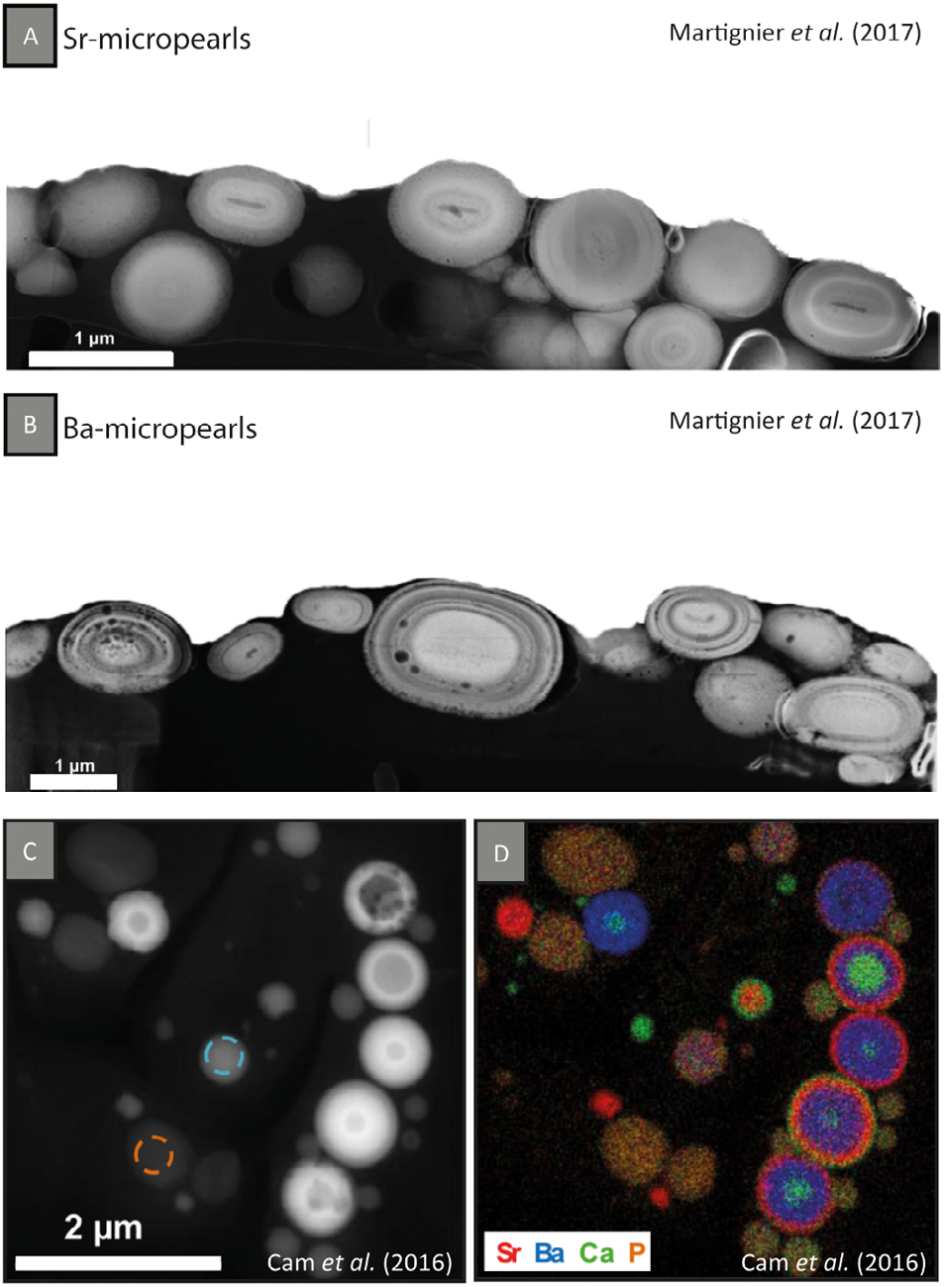
TEM‐HAADF images of FIB‐cut sections of (A) intracellular Sr‐rich micropearls of *T*. *cordiformis* and (B) intracellular Ba‐rich micropearls of a ‘Ba‐organism’. Zonations (concentric layers) can be appreciated in both types of micropearls due to variations in the atomic masses. Darker nuclei are more easily observed in the Sr‐rich inclusions. (C) STEM‐HAADF image of *G*. *lithophora* cells showing the layered structure of the ACC inclusions, with (D) its respective EDXS elemental map showing the variation in chemical composition of the internal layers of the inclusions. Ca appears in green, Sr in red, Ba in blue and P in orange. The images are adapted with permission from: (A, B) Martignier *et al*. (2017). Copyright 2016 John Wiley & Sons. (C, D) Cam *et al*. (2016). Copyright 2016 American Chemical Society.

**Fig 3:**
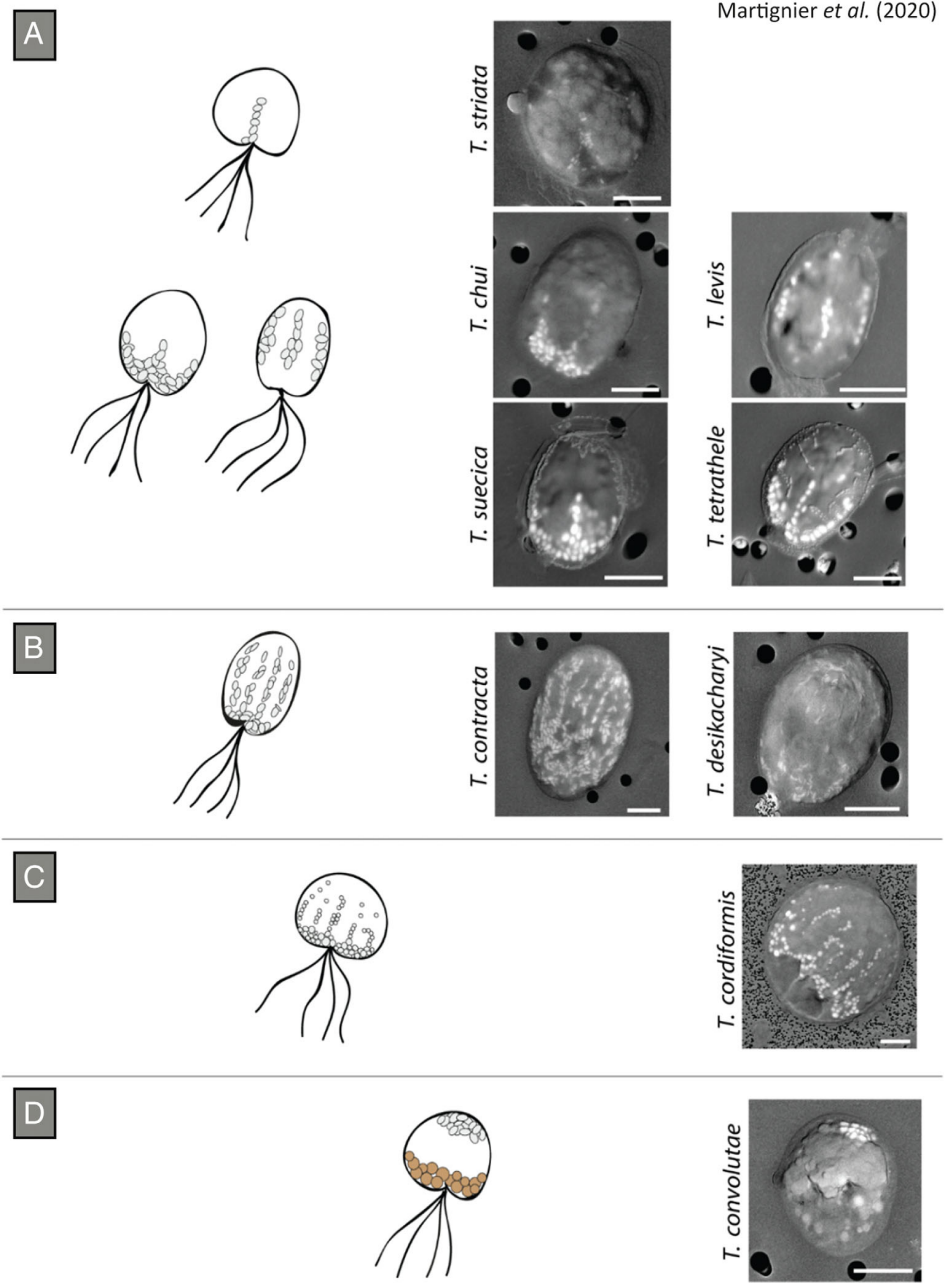
Micropearl distribution patterns vary between the different Chlorodendrophyceae species. The illustrations represent the distribution patterns observed using SEM. (A) A three equidistant lines distribution is observed in the species presenting a four‐lobbed chloroplast (a fourth alignment is hidden at the other side of the cell). (B) Parallel, longitudinal lines of micropearls are distributed throughout the cytoplasm of species having a chloroplast with more than eight lobes. (C) The apical aggregation of micropearls with short longitudinal lines appears in *T*. *cordiformis* due to a net‐like chloroplast. (D) The cup‐shaped chloroplast of *T*. *convolutae* causes an aggregation of micropearls in the posterior pole of the cell. The larger inclusions appearing at the apical pole correspond to phosphate bodies. Scale bars: 5 μm. The figure is adapted with permission from Martignier *et al*. (2020). Copyright 2020 Elsevier.

Transmission electron microscopy (TEM) observation of ultra‐thin cross‐sections of different Chlorodendrophyceae algae shows that the inclusions are located close to the cell surface, under the plasma membrane (Fig. 2A) (Martignier *et al*., 2017, 2018), while SEM observation reveals different distribution patterns of the micropearls between species (Figs 1B, C and 3). A relationship between the chloroplast morphology and the distribution pattern of micropearls has recently been highlighted (Martignier *et al*., 2020): species having a common distribution pattern of micropearls share the same chloroplast morphology. The four‐lobed chloroplast of *T*. *striata*, *T*. *chui*, *T*. *levis*, *T*. *suecica* and *T*. *tetrathele* explains the four longitudinal alignments of micropearls appearing at the apical pole of the cell (observed as three equidistant lines in the SEM because one line is hidden at the other side of the cell) since the inclusions are occupying the interlobular spaces of the chloroplast (Fig. 3A). *T*. *desikacharyi* and *T*. *contracta* present a chloroplast with more than eight lobes, explaining the arrangement of micropearls forming numerous parallel longitudinal lines distributed throughout the cytoplasm (Fig. 3B). The freshwater species *T*. *cordiformis* has a net‐like chloroplast that causes both the apical grouping of the micropearls and their dispersion within the cell (Fig. 3C). Finally, micropearls are clustered at the posterior pole of *T*. *convolutae* cells due to the cup‐like shape of their chloroplast (Fig. 3D).

There also appears to be a relationship between the habitat of the organisms and the shape and distribution pattern of the micropearls (Martignier *et al*., 2018, 2020). Strains sampled in the sand of a marine estuary (*T*. *contracta* and *T*. *desikacharyi*) show a similar distribution pattern of the micropearls (Fig. 3B) that differs from that observed in seawater, free‐swimming strains (*T*. *chui*, *T*. *suecica*, *T*. *tetrathele*, etc.) (Fig. 3A). In addition, while freshwater species present spherical micropearls (Figs 1B, C and 3C), seawater strains form elongated micropearls similar to rice grains (Fig. 3A and B). Chlorodendrophyceae strains not forming micropearls (*T*. *ascus* and *T*. *marina*) tend to be colonial and form sessile structures.

SEM observation of the organisms forming Ba‐rich ACC inclusions in Lake Geneva indicates that they are unicellular eukaryotic organisms with cell sizes varying between 10 and 20 μm and two flagella emerging from one cell pole (Fig. 1A). The smaller cells present micropearls with higher concentrations of Ba (Martignier *et al*., 2017).

### Internal structure and composition of ACC inclusions in protists

TEM‐energy‐dispersive X‐ray spectroscopy analyses of ultra‐thin cross‐sections of Ba and Sr‐rich micropearls, obtained by focused ion beam technology (FIB), reveal internal concentric zonation (nanometric concentric layers of different atomic masses) corresponding to variations in the Sr/Ca and Ba/Ca ratios around a rod‐shaped nucleus (Fig. 2A and B). In both types of organisms, selected area electron diffraction (SAED) and electron energy loss spectroscopy confirm that micropearls are composed of amorphous carbonate (Martignier *et al*., 2017, 2018). In addition, the electron beam triggers bubble formation in the micropearls, suggesting the evaporation of a fluid such as water. Therefore, the composition of micropearls can be related to hydrated amorphous carbonates that are enriched in AEM such as Sr or Ba. High‐resolution elemental mapping using nanoscale secondary ion mass spectrometry (nanoSIMS) shows the presence of S in the organic matter at the surface of both types of micropearls, probably due to the presence of proteins. Carbon‐nitrogen (CN) and S signals are higher in the surrounding area of Sr micropearls than in Ba inclusions, suggesting different biomineralization mechanisms (Martignier *et al*., 2017, 2018).

Finally, SEM‐energy‐dispersive X‐ray spectroscopy analyses also show different Sr accumulation capacities between *Tetraselmis* strains: *T*. *desikacharyi* micropearls present the highest Sr/Ca ratio compared with the culture medium composition (>300 times higher), while *T*. *contracta* and *T*. *cordiformis* are the strains showing the lowest Sr accumulation capacities (Martignier *et al*., 2018).

## Intracellular ACC inclusions in prokaryotes

### Cyanobacteria

In a parallel research line, the use of electron microscopy for the study of biofilms associated with modern microbialites from the hyperalkaline Lake Alchichica (Mexico) had revealed in 2012 the presence of intracellular ACC bioprecipitates in the early‐branching microbialite cyanobacterium *Gloeomargarita lithophora* (order Gloeobacterales) (Couradeau *et al*., 2012). This finding provided the first evidence of a biologically controlled mineralization of calcium carbonate in the intracellular space of cyanobacteria. Interestingly, these carbonates also contained other AEM such as Mg, Sr and Ba. In 2014, SEM observation of cyanobacteria of all the subsections (I, II, III, IV and V) established by Rippka *et al*. (1979) showed that intracellular biomineralization of ACC is a widespread phenomenon in cyanobacteria, including the strain *Candidatus* Synechococcus calcipolaris, also isolated from Lake Alchichica (Benzerara *et al*., 2014).

ACC precipitates of *G*. *lithophora* were observed using SEM (in secondary electron and backscattered electron modes) as bright and spherical inclusions with an average diameter of 270 ± 44 nm (Fig. 1D) (Couradeau *et al*., 2012). There were 21 ± 5 inclusions per cell that occupied 6% of the total cell volume, increasing by 12% the cell density. In most of the cells, the inclusions were scattered throughout the cytoplasm; however, sometimes they appeared to be aligned, forming one or several chains (Li *et al*., 2016). The phylogenetically remote cyanobacteria *Cyanothece* sp. PCC 7425 and *Chroococcidiopsis thermalis* PCC 7203, both obtained from a culture collection, also presented ACC inclusions with a shape and a distribution pattern similar to those observed in *G*. *lithophora* (Benzerara *et al*., 2014). More recently, other strains close to *Cyanothece* sp. PCC 7425 and *Chroococcidiopsis thermalis* PCC 7203 have been also discovered to form this type of ACC inclusions: *Cyanothece* sp. PCC 8303, PCC 8905 and PCC 9303, and *C*. *thermalis* PCC 7432, PCC 7433, PCC 7434, PCC 7439 and PCC 9819 (De Wever *et al*., 2019).

The strains *Ca*. S. calcipolaris G9, *Synechococcus* sp. PCC 6312, *S*. *lividus* PCC 6716 and PCC 6717 and *Thermosynechococcus elongatus* BP‐1 (all from the same ancestral lineage) presented a different distribution pattern of intracellular ACC inclusions, lying at the cell poles and, sometimes, in the middle of the cells (Fig. 1E) (Benzerara *et al*., 2014). The number of inclusions per cell pole varied from 5 to 40 and the diameter of the inclusions from 90 to 300 nm. The inclusions located in the middle of the cell had a diameter comprised between 50 and 150 nm and they seemed to be related to the formation of the cell division septum (Benzerara *et al*., 2014). Similar ACC inclusions have also been found in *Synechococcus* sp. PCC 6603 and PCC 6715 (De Wever *et al*., 2019).

#### Internal structure and composition of ACC inclusions in cyanobacteria

SAED showed the amorphous structure of the inclusions of *G*. *lithophora*, and EDXS analyses indicated a chemical composition similar to the mineral benstonite ((Sr_1_Ba_2.7_Mg_1.4_Ca_0.9_)Ca_6_Mg(CO_3_)_13_). Ba/Ca and Sr/Ca atomic ratios were respectively 1370 and 86 times higher than in the growth medium, suggesting the implication of active import mechanisms to selectively concentrate these elements (Couradeau *et al*., 2012). Indeed, in a culture medium containing a relatively high concentration of Ca and a low concentration of Sr and Ba, *G*. *lithophora* preferentially accumulates Ba in the intracellular ACC inclusions, followed by Sr and Ca (Cam *et al*., 2016). The different AEM contained in the ACC inclusions are frequently deposited sequentially, forming concentric layers (Fig. 2C and D). The inner part of the inclusions (called *core*) can be composed of pure Ca, pure Ba, or almost pure Sr compounds, while the outer part (called *shell*) can be composed of either one or more layers, each enriched in one of these elements.

The incorporation of Ca, Sr and Ba by ACC‐forming cyanobacteria is also reflected in the variation of the chemical composition of the culture medium in which they grow (Cam *et al*., 2016; Cam *et al*., 2018; Blondeau *et al*., 2018a). In *G*. *lithophora* cultures with an initial concentration of 250 μM of Ca, 50 μM Sr and 50 μM of Ba, a decrease of Ba in solution is noticed first, followed by the decrease of Sr and finally of Ca. However, *Cyanothece* sp. does not show the selective incorporation of Sr and Ba under the same culture conditions as *G*. *lithophora*, and the layered structure of the carbonate inclusions is not observed, suggesting that the preferential uptake of Sr and Ba is not a common feature to all ACC‐forming cyanobacteria (Cam *et al*., 2016).

### Alphaproteobacteria and Gammaproteobacteria

A recent study also attributes the ability to form intracellular ACC inclusions to an undescribed genus of magnetotactic bacteria within the class Alphaproteobacteria (Monteil *et al*., 2020), a phylogenetically distant group from cyanobacteria. The studied strain was isolated from the sediment–water interface of Lake Pavin (France) and presents intracellular ACC inclusions in addition to magnetosomes (Fig. 1F). The inclusions occupy most of the cytoplasm and also contain Sr and Ba.

Surprisingly, it has also been shown that the intracellular inclusions of calcium carbonate contained in the well‐known gammaproteobacterium *Achromatium* are composed of ACC and not calcite as commonly thought (Benzerara *et al*., 2020). *Achromatium* cells were also collected from Lake Pavin and the mineral inclusions were analysed using Raman microspectroscopy and SEM. The inclusions present a laminated structure (Yang *et al*., 2019) that could be similar to the layered structure observed in the ACC inclusions of unicellular eukaryotes (Martignier *et al*., 2017) and cyanobacteria (Cam *et al*., 2016).

## Formation of intracellular ACC inclusions in prokaryotes and unicellular eukaryotes

Studies involving the production of synthetic hydrated ACC suggest a spontaneous dehydration of this polymorph in aqueous environments, leading to the formation of calcite, aragonite and/or vaterite crystals (Radha *et al*., 2010; Rodriguez‐Blanco *et al*., 2011; Bots *et al*., 2012). The accommodation of impurities (additives) in the mineral structure enhances the thermal stability of ACC by avoiding dehydration (Goodwin *et al*., 2010; Albéric *et al*., 2018). Hence, ACC might be stabilized in biological environments by the presence of organic macromolecules (such as polysaccharides or proteins) and the addition of some inorganic compounds (such as Mg and phosphate) (Taylor *et al*., 1993; Mann, 2001; Levi‐Kalisman *et al*., 2002; Gal *et al*., 2012). In addition, the isolation of ACC in closed systems (e.g. intracellular vesicles) may also contribute to its stabilization (Cam *et al*., 2015; Mavromatis *et al*., 2017; Martignier, 2019; Benzerara *et al*., 2020).

In microorganisms, the biochemical mechanisms explaining the formation and stabilization of intracellular ACC inclusions are still unknown. This is also the case for the selective incorporation of AEM achieved by several of the unicellular organisms producing these inclusions. An abiotic theory concerning the formation of Ba and Sr‐rich sulfate inclusions in the green alga *Closterium moniliferum* suggests the preferential uptake of Ba and Sr over Ca due to the sulfate‐trap model (Krejci *et al*., 2011). This abiotic model explains preferential precipitation of (Ba,Sr)SO_4_ due to its lower solubility compared with that of SrSO_4_ and CaSO_4_. However, a process in which the formation of the inclusion implies a biological control and a selective active import of Ba and Sr seems more realistic regarding intracellular ACC inclusions (Cam *et al*., 2016). Some ACC‐forming microorganisms have been observed in undersaturated solutions, which is an unfavourable condition to precipitate carbonates (Cam *et al*., 2018). Therefore, the formation of ACC is most likely an active process (costing energy), allowing the supersaturation of the intracellular solution with AEM carbonates (Cam *et al*., 2015; Li *et al*., 2016; Martignier *et al*., 2017; Görgen *et al*., 2021). A biotic–abiotic mixed model has been suggested regarding micropearls formation in Chlorodendrophyceae algae; it combines intracellular pre‐concentration of Sr and Ba with a solid solution growing mechanism (Thien *et al*., 2017). Oscillatory zoning, whereby chemical composition varies more or less regularly along a core‐to‐rim profile, is a well‐known characteristic of solid solution growth (Prieto *et al*., 1997).

Biomineralization of micropearls in at least two different protists seems to start with a rod‐shaped nucleus probably composed of organic matter (Fig. 2A and B) (Martignier *et al*., 2018). In cyanobacteria, it has been proposed that the organization and defined distribution patterns of Ca inclusions in the cytoplasm may also involve a control by the cytoskeleton, which could be implicated in nucleation mechanisms leading to the formation of the inclusions (Benzerara *et al*., 2014; Li *et al*., 2016).

**Fig 4:**
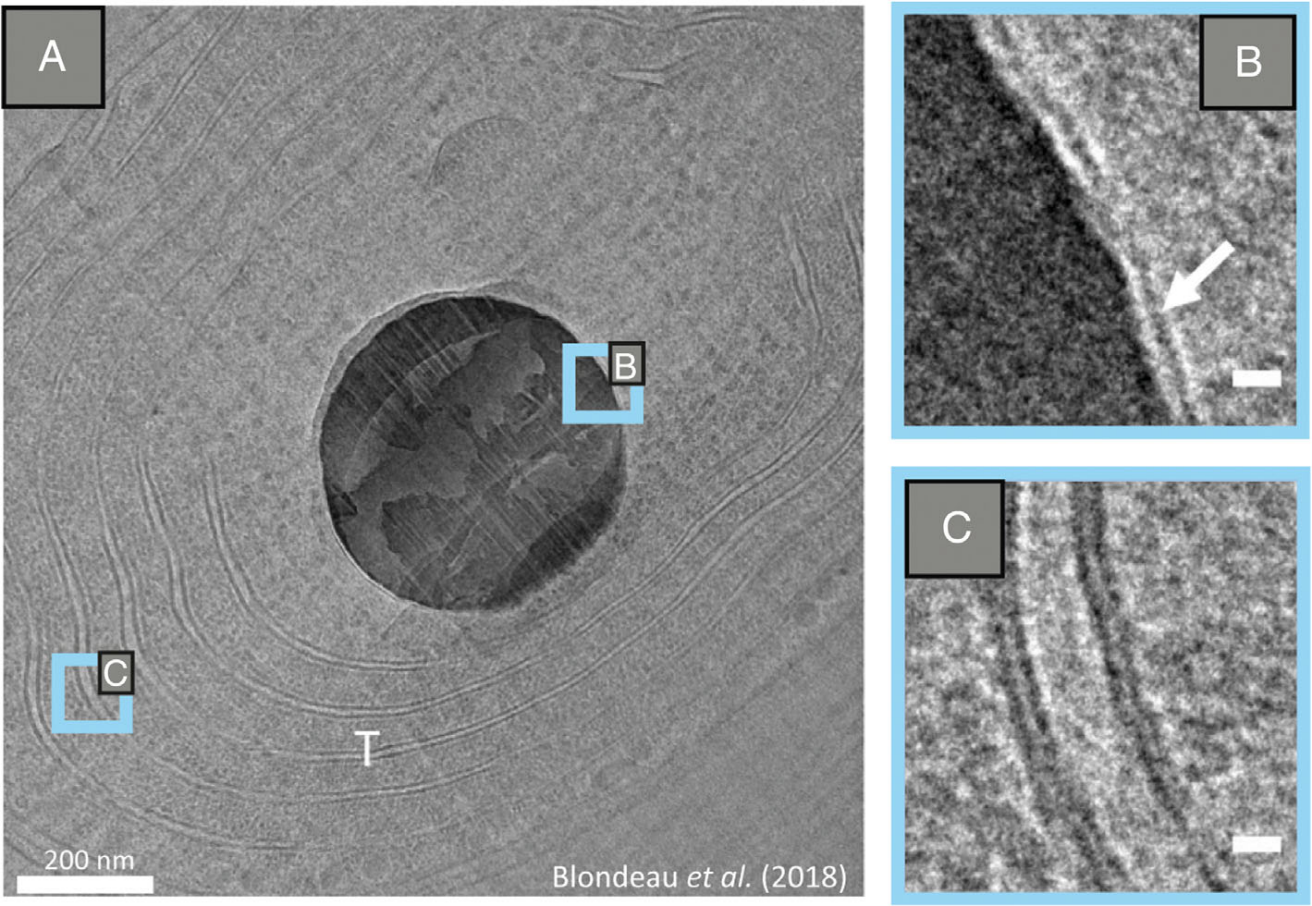
(A) CEMOVIS image of a *Cyanothece* sp. cell with an ACC inclusion in the centre and thylakoids (T). (B) Zoom‐in of the inclusion contour showing a single layer that surrounds the inclusion. (C) Zoom‐in of the lipid bilayer of the thylakoids. Scale bars: (B, C) 0.5 nm. The images are adapted with permission from Blondeau *et al*. (2018b). Copyright 2018 Blondeau, Sachse, Boulogne, Gillet, Guigner, Skouri‐Panet, Poinsot, Ferard, Miot, and Benzerara.

The formation of ACC inclusions in cyanobacteria has been suggested to take place in intracellular vesicle‐like structures, avoiding direct contact with the cytoplasmic solution that could affect the stability of the inclusions (Cam *et al*., 2015). In a recent study using both freeze‐substitution sample preparation and cryo‐electron microscopy of vitreous sections (CEMOVIS) technologies, Blondeau *et al*. (2018b) demonstrate the presence of ~2.5 nm thick structures of unknown nature around the ACC inclusions of cyanobacteria (Fig. 4). Two hypotheses are proposed regarding the nature of these structures: they are either protein shells or lipid monolayers. In the case of the Alphaproteobacterium undescribed genus from Lake Pavin, a lipid bilayer surrounding the ACC precipitates has been noticed using CEMOVIS (Monteil *et al*., 2020). It has also been suggested that the ACC granules in *Achromatium* are confined by lipid bilayers (Gray and Head, 2014; Benzerara *et al*., 2020). Although these structures have not yet been observed in microalgae, the occurrence of S and CN surrounding the micropearls could indicate the presence of a proteinaceous layer (Martignier *et al*., 2017).

Recently, at least three types of genes have been detected in all the cyanobacteria strains presenting ACC inclusions: a gene encoding for a mechanosensitive channel that is probably involved in Ca import, a Ca^2+^/H^+^ transporter and UPF0016 membrane proteins that may be related to the active transport of Ca (De Wever *et al*., 2019). However, these genes do not appear to be specific to ACC‐forming cyanobacteria, as similar genes could be found in other species that do not form intracellular ACC (De Wever *et al*., 2019). Thus, future studies are needed to decipher the molecular mechanisms behind the formation of these intracellular inclusions.

## Physiological role of ACC inclusions

ACC has been found in several phyla (Plantae, Porifera, Cnidaria, Echinodermata, Mollusca and Arthropoda) and can constitute temporary storage deposits, a stiffener of certain tissues, or a precursor phase of a crystalline mineral (Addadi *et al*., 2003; Radha *et al*., 2010). A study of extracellular ACC inclusions in *Ficus* leaves called *cystoliths* indicated that this mineral could also be involved in the regulation of intracellular pH (Gal *et al*., 2012).

The biological functions of ACC inclusions in bacteria and microalgae are still largely unknown. However, when cultured in a growth medium with an initial concentration of 50 μM of Ca, the growth rate of ACC‐forming cyanobacteria is limited compared with cultures with an initial concentration of 250 μM of Ca. Moreover, after 527 h of culture, the addition of 200 μM of Ca in the cultures with an initial Ca deficit (50 μM) shot up the growth rate. In non‐ACC‐forming cyanobacteria, the growth is barely affected by an initial Ca deficit. Hence, it has been suggested that a high Ca accumulation is essential for the growth of ACC‐forming cyanobacteria (De Wever *et al*., 2019). Nevertheless, it is still unclear whether such high Ca uptake is necessary for certain biochemical processes or needed because ACC inclusions fulfil an important function in cyanobacteria. Several studies have postulated different hypotheses related to the biological functionality of ACC inclusions in cyanobacteria: (i) they may have a role linked with buoyancy, acting as ballast by increasing the cell density as an adaptation to benthic life (Couradeau *et al*., 2012; De Wever *et al*., 2019). (ii) They could constitute intracellular deposits where excess alkalinity produced during photosynthesis is stored rather than being transported extracellularly (Couradeau *et al*., 2012). (iii) They could serve as inorganic carbon deposits when their availability in the environment is limited (De Wever *et al*., 2019). (iv) They could buffer the intracellular pH (Couradeau *et al*., 2012; De Wever *et al*., 2019). (v) They could be related to cell division since the inclusions in *Synechococcus* sp. PCC 6312 appear in the septation site (Benzerara *et al*., 2014).

In the newly discovered genus of Alphaproteobacteria, ACC inclusions represent an important increase of the cell density since they can occupy two‐thirds of the cytoplasm. This may explain the gravitaxis observed in this type of organism, allowing cells to adjust their position at the bottom of the water column (Monteil *et al*., 2020). In the giant gammaproteobacterium *Achromatium*, the ACC inclusions could buffer the intracellular pH, avoiding important pH variations due to the redox transformations of S species (Yang *et al*., 2019; Benzerara *et al*., 2020).

In Chlorodendrophyceae algae, micropearls may constitute Ca reserves that could have a biological role in theca and scales formation, flagella renewal, buoyancy and motility (Martignier *et al*., 2020). In 1982, Ca‐sequestering vesicles with diameters from 0.25 to 0.5 μm were detected in the anterior part of *Tetraselmis subcordiformis* by using calcium pyroantimonate and calcium oxalate cytochemistry, and energy‐dispersive X‐ray microanalyses (Salisbury, 1982). The vesicles were suggested to rise free Ca^2+^ in the cytosol, triggering the contraction of the striated flagellar roots (contractile structures associated with the basal bodies of flagella that act as a ‘rudder’), and changing the swimming direction of the cells (Salisbury, 1982; Salisbury *et al*., 1984). The cellular location, size and shape of the Ca sequestering vesicles seem to match the characteristics of micropearls in *Tetraselmis*, as they are usually located in the anterior part of the cells and have an average length of 0.4 μm in *T*. *subcordiformis* (Martignier *et al*., 2018, 2020).

## Habitats

ACC‐forming microorganisms are varied and inhabit multiple different habitats. The existing studies suggest that they tend to colonize environments with dissolved Ca available (Table 1). The micropearls produced by *T*. *cordiformis* and the ‘Ba‐organisms’ were observed for the first time in Lake Geneva, where the surface water is oversaturated with calcite between April and August (Jaquet *et al*., 2013). However, most of the Chlorodendrophyceae strains studied have also been obtained from algae collections and cultured in diverse growth media (Martignier *et al*., 2018). Chlorodendrophyceae are widespread in the world and colonize different habitats, from freshwater (*T*. *cordiformis* and *Scherffelia dubia*) to brackish water and seawater (*T*. *chui*, *T*. *suecica*, *T*. *tetrathele*, etc). Lifestyles are also varied among the different strains: they can be found in the sediments (i.e. *T*. *contracta*) or the water column (i.e. *S*. *dubia*, *T*. *chui*, *T*. *suecica*, *T*. *tetrathele*, etc), as temporary sessile colonies or as free‐swimming organisms (Martignier *et al*., 2018, 2020). *Tetraselmis convolutae* can also be found as a photoendosymbiont under the epidermis of the flatworm *Symsagittifera roscoffensis* (Selosse, 2000).

**Table 1:** Comparison of the physicochemical conditions of water at GE3 station in Lake Geneva (46.2994 °N/6.2197 °E) and Lake Alchichica aquarium.

Study	Location	Date	*T* (°C)	pH	Alkalinity (μM L^−1^)	Mg (μM L^−1^)	Ca (μM L^−1^)	Sr (μM L^−1^)	Ba (μM L^−1^)	*Saturation Index* Calcite
Jaquet *et al*. (2013)	Lake Geneva, GE3 station (5 m depth)	01/07/2012	16	8.14	1730	235	1004	5.3	0.12	0.13
		05/08/2012	19.4	8.38	1560	232	924	5	0.11	0.34
Couradeau *et al*. (2012)	Alchichica aquarium		24	8.9	13 617	6955	47	0.013	0.001	0.158

The indicated values for Lake Geneva correspond to those obtained at 5 m depth, where micropearls were more abundant. Both environments present a basic pH and a similar saturation index (*SI*) of calcite based on the formula: *SI* = log (*IAP/K*_*s*_).

Besides the two strains isolated from the alkaline Lake Alchichica (*G*. *lithophora* and *Ca*. S. calcipolaris), most of the cyanobacteria known today to form carbonate inclusions were obtained from culture collections (in particular, from the Pasteur Culture Collection of Cyanobacteria). The origin of the collected strains was very diverse: from German soils to rice fields in Senegal and hot springs in the USA and Japan (Benzerara *et al*., 2014). Interestingly, members of the *G*. *lithophora* and *Ca*. S. calcipolaris lineage have also been identified in microbialites of other Mexican lakes close to Lake Alchichica, as well as in karstic areas and microbial mats collected in South America and/or Southern Europe (Ragon *et al*., 2014). The undescribed genus of magnetotactic bacteria within the class Alphaproteobacteria and the giant sulfur bacteria *Achromatium* presenting ACC inclusions were both collected from the ferruginous and meromictic Lake Pavin (Monteil *et al*., 2020). The magnetotactic bacterium was collected from the sediments and the water column (Monteil *et al*., 2020), while *Achromatium* was collected from the sediments and the water overlaying them (Benzerara *et al*., 2020). In addition, unidentified cells containing ACC inclusions have also been observed in the oxic water of Lake Pavin (Miot *et al*., 2016).

## Impact on the geochemical cycles

ACC‐forming organisms, and especially those concentrating Ba and Sr, may have an unexpected impact on the geochemical cycle of AEM. For instance, an experiment in which the cyanobacterium *G*. *lithophora* is cultured in a growth medium supplemented with carbonate microbialite fragments (that act as a continuous source of Ca, Sr, and Ba in the medium) shows the capacity of this organism to buffer the dissolved Ba/Ca and Sr/Ca ratios at low values (Blondeau *et al*., 2018a). Additionally, it has been proposed that the presence of microorganisms forming Ba‐rich micropearls in Lake Geneva could have an impact on the geochemical cycle of Ba in this lake since an inverse correlation was observed between the number of ‘Ba‐organisms’ and the concentration of soluble Ba in the lake waters (Martignier *et al*., 2017).

Thus, these organisms have been suggested as plausible candidates for the development of new bioremediation techniques regarding Ba and radioactive ^90^Sr pollution (Cam *et al*., 2016; Martignier *et al*., 2017, 2018; Blondeau *et al*., 2018a). The cyanobacterium *G*. *lithophora* has been shown to efficiently sequester ^226^Ra and ^90^Sr when these radionuclides were added to the culture medium (Mehta *et al*., 2019). Nevertheless, more in‐depth studies are still needed to better understand the processes involved in the incorporation of these elements in order to critically assess any possible use of these organisms for bioremediation purposes.

## Why have ACC inclusions only recently been observed?

The ultrastructure of several strains of cyanobacteria known today to form ACC inclusions had been studied in the past but no description of the inclusions was published (Allen, 1984; Porta *et al*., 2000; Blondeau *et al*., 2018b). The same applies to Chlorodendrophyceae algae (Melkonian, 1979; Hori *et al*., 1982), commonly studied because of their interest in aquaculture (Gladue and Maxey, 1994; Meseck *et al*., 2005). Only some early microscopists had noticed the ACC inclusions as refractive inclusions but none had described their chemical composition (Carter, 1937; Hollande *et al*., 1954; Butcher, 1959; Norris *et al*., 1980; Martignier *et al*., 2020).

It has been noticed that ACC inclusions are translucent and very sensitive to pH variation. Therefore, they have probably been overlooked in optical microscope observations and dissolved during sample preparation for electron microscopy, in which chemical fixatives are normally used (Li *et al*., 2016; Martignier *et al*., 2017, 2018; Schorn *et al*., 2020). ACC inclusions from Lake Alchichica and Lake Geneva were observed for the first time using SEM because the organisms were filtered and dried without using any chemical fixatives (Couradeau *et al*., 2012; Jaquet *et al*., 2013). The use of buffers (such as cacodylate buffer 2%) during the fixation process (Martignier *et al*., 2017) and the application of other techniques such as freeze‐substitution and CEMOVIS (Blondeau *et al*., 2018b) also allow the preservation of ACC inclusions for SEM and TEM observation. Differential interference contrast (DIC) microscopy also allows the observation of the micropearls in living Chlorodendrophycea algae (Martignier *et al*., 2020). ACC inclusions in *Achromatium* have been shown to be sensitive to strong laser irradiation (e.g. during Raman microspectroscopy analyses), which causes their spontaneous transformation into calcite (Benzerara *et al*., 2020). Thus, this may explain why the inclusions in *Achromatium* were commonly thought to be composed of calcite rather than ACC.

Finally, the high instability and the amorphous character of the ACC inclusions may prevent their fossilization and therefore their observation in sediments is rather unlikely (Riding, 2012; Cam *et al*., 2018).

## Evolutionary theories of ACC‐forming organisms

**Fig 5:**
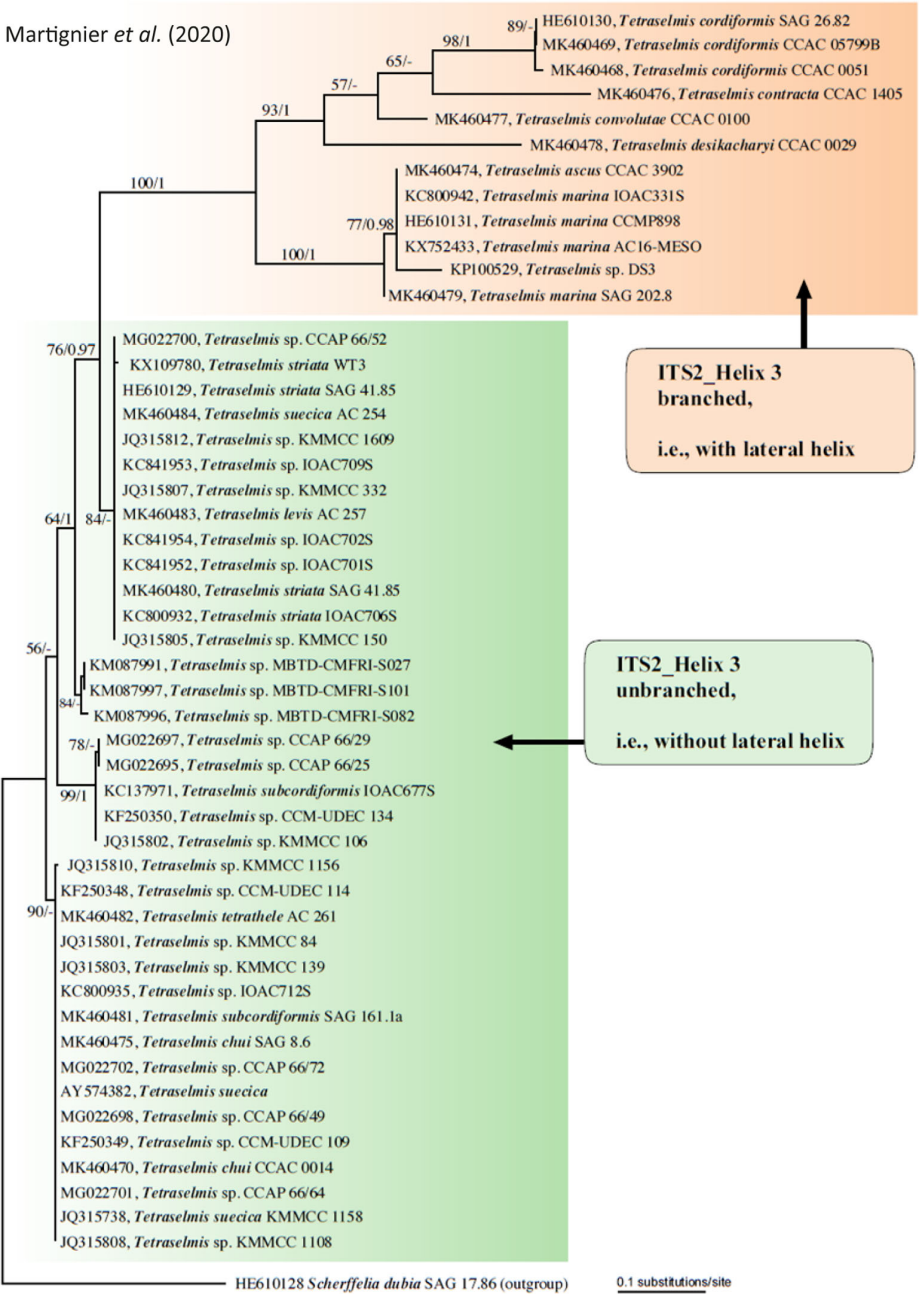
Phylogeny of Chlorodendrophyceae strains based on 5.8S rDNA and ITS2 sequence data. The green cluster includes *Tetraselmis* strains presenting four longitudinal alignments of micropearls in the apical pole of the cell. The orange cluster includes *Tetraselmis* strains presenting other distribution patterns of the micropearls together with the strains not forming micropearls. Genus *Scherffelia* is considered an outgroup. The figure is reprinted with permission from Martignier *et al*. (2020). Copyright 2020 Elsevier.

Cyanobacteria forming intracellular ACC inclusions are diverse and occupy distant positions in the phylogenetic tree, suggesting the existence of an early common ancestor presenting this biomineralization ability (Benzerara *et al*., 2014). This theory is reinforced by the presence of ACC inclusions in *G*. *lithophora* and *T*. *elongatus* BP‐1, considered as deep‐branching cyanobacteria (Couradeau *et al*., 2012; Benzerara *et al*., 2014). Moreover, all the known strains belonging to the *T*. *elongatus* BP‐1 clade form intracellular ACC inclusions, showing that this biomineralization process can be heritable (Benzerara *et al*., 2014; Görgen *et al*., 2021). The idea of a common ancestor could also be valid regarding the two genera of microalgae within the Chlorodendrophyceae class (*Tetraselmis* and *Scherffelia*) since both genera form micropearls (Martignier *et al*., 2020). Adaptation to different ecological niches could have led to differentiation in the shape and distribution of micropearls between the Chlorodendrophyceae strains, influenced by the morphological changes of chloroplasts (Martignier *et al*., 2020). Analysis of the 5.8S/ITS2 and the *rbc*L sequences revealed the phylogenetic proximity of the strains sharing the same distribution pattern of micropearls, indicating that the micropearls arrangement could be used as a criterion to identify different phylogenetic groups within the class Chlorodendrophyceae (Martignier *et al*., 2020). Two main groups were set up according to the structure of the ITS2 helix. The first group was composed of all the *Tetraselmis* strains presenting four longitudinal alignments of micropearls in the apical pole of the cell, and the second one included *Tetraselmis* strains presenting other distribution patterns of micropearls together with the strains not forming micropearls (Fig. 5). Genus *Scherffelia* was defined as an outgroup even if this strain also presents a four‐lobbed chloroplast, suggesting that *Scherffelia* is the oldest divergence in the class.

The eukaryotic organisms forming intracellular Ba‐rich ACC inclusions observed in Lake Geneva remain a mystery and not much can be said about their phylogenetical position. However, there are certain similarities between these organisms and the ciliates described by Fauré‐Fremiet and Gauchery (1957), some of them presenting intracellular spherical granules of calcium carbonate that may be in an amorphous state associated with organic matter (e.g. *Loxocephalus granulosus*).

Concerning the magnetotactic bacteria collected from Lake Pavin, no other descriptions of ACC inclusions have been made within the class Alphaproteobacteria (Monteil *et al*., 2020). Nonetheless, two strains of magnetotactic bacteria within the class Gammaproteobacteria have been reported to possess Ca‐rich inclusions: (i) the giant rod‐shaped magnetotactic bacterium GRS‐1 (Taoka *et al*., 2014) and (ii) a magnetotactic bacterium obtained from the Seine River (France) (Isambert *et al*., 2007). Nevertheless, it is still yet unknown whether these inclusions are in an amorphous phase or not (Monteil *et al*., 2020). The anoxic sulfur bacterium *Achromatium* is the only genus included in the class Gammaproteobacteria that is known to date to form intracellular ACC inclusions (Benzerara *et al*., 2020). Therefore, it is challenging to know whether or not these groups of bacteria are related by a common ancestor able to form intracellular calcium carbonate inclusions.

In brief, the newly discovered biomineralization process involved in the formation of intracellular ACC inclusions seems to be similar in all the groups of microorganisms producing them. However, these groups are probably too distant in the phylogenic tree to consider an evolutionary link between all of them. Therefore, the intracellular biomineralization phenomenon of ACC has probably appeared in such different groups of organisms due to a convergent evolution (independent evolution resulting in common features), relying on different molecular processes. Then, this biomineralization capacity may have been subsequently transmitted to species included in the same clade through a common ancestor. For instance, this could explain why ACC inclusions in microalgae have only been observed in the class Chlorodendrophyceae, and why some differences in the shape and distribution patterns of micropearls have been noticed between species, as a result of adaptation to different environments (divergent evolution) (Martignier *et al*., 2020). However, further phylogenetic analyses still need to be carried out to clarify any possible evolutionary relationship of all these organisms.

## The way ahead

Future research efforts to describe the biomineralization processes leading to the formation of intracellular ACC inclusions in microorganisms are needed to answer unresolved questions. In‐depth analyses of the variations in the chemical composition of the media where the ACC‐forming microorganisms are cultured will provide additional information on the AEM uptake kinetics and their impact on the geochemical cycles. Moreover, additional experiments involving cultures with added ^90^Sr remain crucial to investigate the feasibility of using ACC‐forming microorganisms for the development of new bioremediation methods. Further comparative genomic analyses are necessary to identify the molecular mechanisms involved in the formation and stabilization of the ACC inclusions. Experiments involving isotope labelling could also provide insight into the cellular pathways related to their formation. In addition, the detection of culture conditions affecting the formation of the ACC inclusions could shed light on their biological role. Finally, future molecular phylogenetic studies are essential to understand the origin of these processes and their diversification among microorganisms.

## Concluding remarks

Originally, the biomineralization of calcium carbonate was mainly thought to be an extracellular process in microorganisms; however, recent studies have shown that the formation of intracellular ACC inclusions is a widespread phenomenon in both prokaryote and eukaryote unicellular organisms. These inclusions can be observed using SEM and TEM, with an appropriate preparation and DIC optical microscopy for living organisms. Many of the organisms possessing this newly discovered biomineralization capacity can concentrate Ba and/or Sr and may thus have an impact on the geochemical cycle of AEM. This peculiar feature makes these microorganisms to be considered interesting candidates to develop new bioremediation technologies regarding Ba and radioactive ^90^Sr pollution, also because they can be found in very different living environments. The biological functionality of the ACC inclusions, as well as the molecular processes involved in their formation, is not clear yet and require more in‐depth research. The occurrence of intracellular ACC inclusions in phylogenetically distant groups of microorganisms suggests an independent evolution leading to similar biomineralization processes.
